# The impact of physical activity on subjective wellbeing in college students: the chain-mediating role of rumination and mindfulness

**DOI:** 10.3389/fpsyg.2025.1575612

**Published:** 2025-08-18

**Authors:** Shimeng Wang, Bochun Lu, Xinming Zhang

**Affiliations:** Institute of Sports Science, Nantong University, Nantong, China

**Keywords:** physical activity, subjective wellbeing, rumination, mindfulness, chain mediation, mental health intervention

## Abstract

**Background:**

Subjective wellbeing (SWB) is an important indicator of mental health, and physical activity (PA) has been shown to have a positive impact on SWB. However, the specific pathways through which PA enhances SWB via psychological mechanisms remain unclear. Cognitive regulation theory suggests that emotional experiences are influenced by cognitive processes, where rumination (RT) and mindfulness (MDS) play key roles in regulating emotional states. This study aims to explore the chain-mediating roles of RT and MDS in the relationship between PA and SWB, in order to deepen the understanding of how PA affects mental health.

**Methods:**

This study used a cross-sectional design and collected data on PA, RT, MDS, and SWB from university students through a questionnaire survey. A total of 1,075 college students (Mage = 19.84, SD = 1.36; 48.9% male) from China participated in the study. Pearson correlation analysis was used to explore the relationships between variables, and PROCESS Model 6 was employed for mediation analysis to examine the chain-mediated effects of PA on SWB through RT and MDS.

**Results:**

The results indicate that PA has a significant direct effect on SWB, and it also influences SWB indirectly through RT and MDS. Specifically, PA reduces RT, and the decrease in RT further enhances MDS. Additionally, PA directly promotes MDS, and higher levels of MDS contribute to higher SWB. These findings suggest that the positive impact of PA on SWB is not only due to emotional regulation but also involves the optimization of cognitive processes, including reducing negative thinking and enhancing positive psychological resources.

**Conclusion:**

This study reveals the chain-mediated mechanism through which PA enhances SWB by reducing RT and increasing MDS. These findings highlight the importance of integrating exercise and cognitive regulation in mental health interventions. The study suggests that combining exercise training and MDS interventions may be a more effective strategy for promoting mental health. Future research should explore how different types of exercise affect MDS and optimize exercise-cognitive interventions for specific populations.

## Introduction

### Background

In modern society, the mental health of college students has become an increasing concern. With rising academic pressure, intensified job market competition, and the growing complexity of social environments, subjective wellbeing (SWB) among college students has shown a declining trend. SWB not only affects an individual's psychological state but also has a direct impact on academic performance, interpersonal relationships, and future career development (Wei and Zhang, 2023). In recent years, researchers in psychology and public health have actively explored effective strategies to enhance students' wellbeing to improve their overall quality of life (Diener et al., 2018). Physical activity (PA) has been widely recognized as a key factor in promoting both physical and mental health (Bouchard et al., 2012). On a physiological level, regular PA enhances cardiovascular function, strengthens the immune system, and reduces the risk of chronic diseases (Anderson and Durstine, 2019; Sothern et al., 1999). On a psychological level, physical exercise facilitates the release of neurotransmitters such as endorphins, dopamine, and serotonin, directly improving mood and reducing anxiety and depressive symptoms (Sothern et al., 1999; Mikkelsen et al., 2017). Additionally, engaging in PA can enhance self-esteem, psychological resilience, and stress management while lowering anxiety sensitivity, thereby fostering overall psychological wellbeing (Zheng et al., 2024; Lin et al., 2024; Smits et al., 2008). However, while previous studies have demonstrated the positive impact of PA on SWB (Buecker et al., 2021), this process is not solely dependent on physiological improvements; rather, it involves complex psychological regulatory mechanisms. Psychological research suggests that SWB is largely influenced by cognitive patterns and emotional regulation abilities (Balzarotti et al., 2016; Korpela et al., 2018). Among these, rumination and mindfulness are critical psychological factors that shape emotional experiences and psychological adaptation.

### The role of rumination

Rumination (RT) is a cognitive pattern that significantly affects individual mental health. It has been extensively studied and is recognized as a core mechanism underlying anxiety and depression (Nolen-Hoeksema et al., 2008). RT refers to the persistent and repetitive focus on one's negative emotions and experiences when facing adverse events, rather than actively seeking solutions or engaging in positive coping strategies (Nolen-Hoeksema et al., 2008). High levels of RT have been empirically linked to depression, anxiety, reduced self-esteem, and maladaptive emotional regulation strategies, playing a crucial role in the development and maintenance of psychological disorders (Kuster et al., 2012; McLaughlin and Nolen-Hoeksema, 2011). Existing research suggests that PA may reduce RT, thereby enhancing SWB (Bernstein and McNally, 2018). This mechanism may involve attention shifting, emotional regulation, and neuroplasticity (Bernstein and McNally, 2018). Firstly, exercise redirects individuals' attention toward bodily sensations and the external environment, reducing excessive focus on negative thoughts and alleviating cognitive load (Zhang et al., 2022). As a result, exercise reduces cortisol levels and triggers dopamine as well as serotonin release, which greatly enhances the ability to regulate emotions and speeds up the recovery from negative emotional states (Bernstein and McNally, 2018). Also, studies with neuroimaging have shown that performing physical exercises can improve the activity of brain areas such as the prefrontal cortex and amygdala which are responsible for regulating emotions, and improve the ability to control cognitive functions and decrease the incidence of involuntary negative thinking (Zhang et al., 2022). Still, most of the available research depicts the repercussions of exercise on emotional regulation rather than the effects of exercise on RT because few studies have aimed to understand this phenomenon. More research is needed to explain how exercise impacts SWB through restorative processes.

### The role of mindfulness

Mindfulness (MDS) is a fundamental cognitive and emotional regulation mechanism that plays a crucial role in enhancing mental health and SWB (Grossman et al., 2004). It is not only a sustained awareness of present experiences but also emphasizes an open, non-judgmental acceptance of one's emotions, thoughts, and external environment (Alvear et al., 2022). Unlike general attention focus, MDS is not merely about being present but also about how individuals interact with their experiences—observing their inner states consciously rather than being dominated by automatic emotional reactions (Kang et al., 2013). Empirical studies have demonstrated that higher levels of MDS are associated with reduced anxiety and depression, as well as improved emotional regulation (Brockman et al., 2017). Research by Brockman et al. suggests that MDS enables individuals to adopt cognitive reappraisal strategies rather than emotional suppression when dealing with stress, thereby preventing the accumulation of negative emotions (Brockman et al., 2017). Furthermore, MDS facilitates both short-term emotional regulation and long-term psychological resilience, allowing individuals to maintain greater psychological flexibility when facing stress and challenges (Brockman et al., 2017). From a neuroscience perspective, MDS practice has been shown to enhance cognitive control by strengthening the prefrontal cortex (PFC) while simultaneously reducing hyperactivity in the amygdala, which is responsible for processing negative emotions (Wheeler et al., 2017). These neural changes indicate that MDS enables individuals to remain calm under stress, reducing automatic emotional reactivity and enhancing overall emotional regulation capacity. Additionally, MDS-based interventions, such as Mindfulness-Based Stress Reduction (MBSR) and Mindfulness-Based Cognitive Therapy (MBCT), have been widely implemented in mental health interventions. These approaches have demonstrated significant efficacy in alleviating anxiety, depression, and chronic pain, as well as in improving SWB (Sado et al., 2020).

### Research objectives and hypotheses

**Figure 1:**
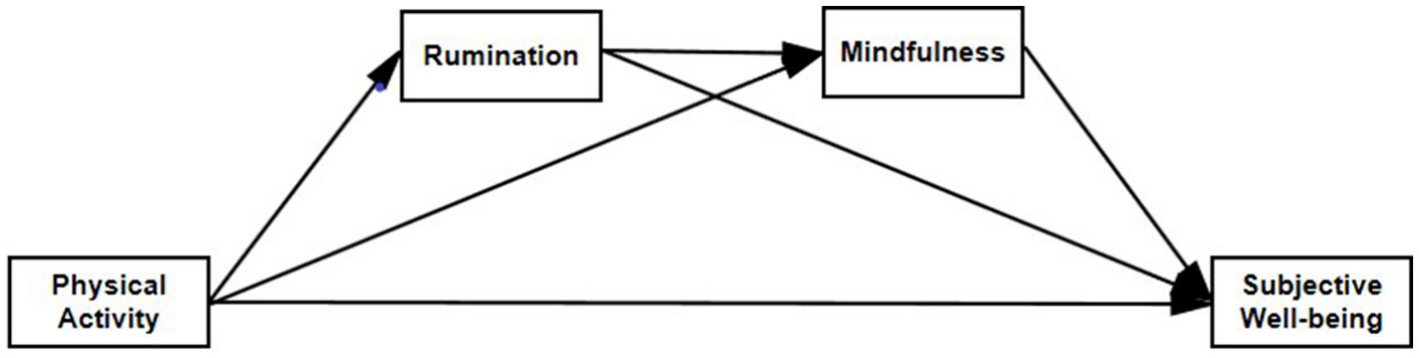
Hypothetical modeling of the path of PA's effect on SWB.

Previous studies have suggested that PA may influence mental health by enhancing MDS levels (Schuch et al., 2018); however, the specific mechanisms linking PA to SWB remain insufficiently explored. From the perspective of cognitive regulation theory, MDS is not only an emotion management strategy but also a key variable that affects an individual's information processing patterns. It may play a chain-mediating role in the relationship between PA and SWB. Specifically, PA may first reduce RT, making it easier for individuals to attain a mindful state. The subsequent increase in MDS could further facilitate the generation of positive emotions and reduce emotional fluctuations, ultimately enhancing SWB. Additionally, existing research has demonstrated that MDS improves self-regulation, allowing individuals to focus on the present moment and reduce automatic negative reactions to distressing experiences. This suggests that MDS may serve as a critical psychological pathway in the chain mediation model. Based on this theoretical framework, this study aims to examine the impact of PA on SWB among college students and further explore the chain-mediating roles of RT and MDS. This research will not only deepen our understanding of the relationships between PA, psychological regulation mechanisms, and SWB but also provide theoretical and practical implications for promoting mental health interventions among college students. Based on these hypotheses, this study proposes a hypothetical model illustrating the relationships among PA, RT, MDS, and SWB. Please refer to Figure 1.

- **H1**: PA positively predicts SWB.
- **H2**: RT partially mediates the relationship between PA and SWB, i.e., PA → RT → SWB.
- **H3**: MDS partially mediates the relationship between PA and SWB, i.e., PA → MDS → SWB.
- **H4**: RT influences SWB through MDS, forming a chain mediation effect, i.e., PA → RT → MDS → SWB.

## Methods

### Data collection and sample selection

This study recruited college students from four universities in Jiangsu Province as participants. Data were collected through an online questionnaire survey administered via Wenjuanxing (https://www.wjx.cn). The survey was conducted from November 1, 2024, to December 1, 2024. Questionnaires were distributed through university administrative departments, class WeChat groups, and QQ groups. A total of 1,200 questionnaires were distributed, all of which were returned, resulting in a 100% response rate. To ensure data quality, the research team conducted a rigorous screening process. Questionnaires with incomplete responses, logical inconsistencies, or extremely short completion times (<3 min) were excluded. Additionally, reverse-coded items were used to check for response consistency, ensuring that participants answered attentively. After data cleaning, 1,079 valid responses were retained, accounting for 89.9% of the total distributed questionnaires. This high-quality dataset provides a reliable foundation for subsequent data analysis (see Table 1).

**Table 1:** Participants characteristic.

**Category**		***N***	**%**
Age (years)	17	11	1.0
	18	448	41.7
	19	399	37.1
	20	199	18.5
	21	18	1.7
Gender	Male	526	48.9
	Female	549	51.1
PA score	Low PA	525	48.8
	Moderate PA	235	21.9
	High PA	315	29.3
Total		1,075	100.0

### Measures and instruments

#### Physical Activity Rating Scale-3

The study adopts the Physical Activity Rating Scale-3(PARS-3) revised by Liang (1994) to assess the level of student's PA. PARS-3 includes three items which are exercise intensity (How intense do you normally exercise?), exercise time (How long do you normally exercise each time?) and exercise frequency (What is the frequency that you exercise in a week/month?). Each item has five response options scored from 1 to 5 points. For example, for the exercise frequency item, the options are: less than once a month (1 point), two to five times a month (2 points), once to twice a week (3 points), three to four times a week (4 points), and every day (5 points). For the exercise intensity item, the options range from very light (1 point), light (2 points), moderate (3 points), somewhat intense (4 points), to very intense (5 points). For exercise time, the options are <10 min (1 point), 10–20 min (2 points), 21–30 min (3 points), 31–60 min (4 points), and more than 60 min (5 points). The PA score is calculated based on this formula: PA Score = Exercise Intensity Score^*^(Exercise Time Score – 1)^*^Exercise Frequency Score. The PA score is ranged between 0 and 100. Meanwhile, according to the PA score, the total PA score is classified into three levels: low (≤ 19 points), moderate (20–42 points), and high (≥43 points) (Liang, 1994).

#### Rumination Response Scale

The Rumination Response Scale (RRS) was originally developed by Nolen-Hoeksema (Nolen-Hoeksema et al., 2008) and later adapted into a Chinese version by Han et al. (Han and Yang, 2009). The scale consists of 22 items and is scored on a 4-point Likert scale, with higher scores indicating a greater tendency toward RT. It measures three dimensions: symptomatic rumination, compulsive thinking, and reflective pondering. Among them, symptomatic rumination includes items 1, 2, 3, 4, 6, 8, 9, 14, 17, 18, 19, and 22; compulsive thinking consists of items 5, 10, 13, 15, and 16; and reflective pondering includes items 7, 11, 12, 20, and 21. The Chinese version of the RRS has been widely validated among Chinese college students and has demonstrated strong construct validity and reliability (Han and Yang, 2009). Its application in previous studies suggests that it is an effective tool for assessing RT tendencies within this population.

#### Five-Factor Mindfulness Questionnaire

The Five-Factor Mindfulness Questionnaire (FFMQ), developed by Baer et al. (2006) was used to assess participants' MDS levels and evaluate changes in their MDS state. This scale consists of 39 items and covers five core dimensions: Observing, Describing, Nonjudging, Nonreactivity to Inner Experience, and Acting with Awareness. In this study, both the total MDS score and subscale scores were calculated, with higher scores indicating higher levels of MDS.The Chinese version of the Five-Factor Mindfulness Questionnaire (FFMQ-C), revised by Deng Yuqin, was used in this study. The revised version has demonstrated good reliability and validity (Deng et al., 2011).

#### Subjective wellbeing

SWB consists of two dimensions: cognitive wellbeing and affective wellbeing (Andrews et al., 1976). The cognitive dimension is assessed using the Satisfaction With Life Scale (SWLS), developed by Diener et al. (1985). This scale measures individuals‘ overall evaluation of their current life satisfaction. The SWLS consists of five items, rated on a 7-point Likert scale, ranging from 1 (“strongly disagree”) to 7 (“strongly agree”). Higher total scores indicate greater life satisfaction. The affective dimension is measured using the Positive and Negative Affect Schedule (PANAS), developed by Watson, Clark, & Tellegen (Andrews et al., 1976). This scale assesses individuals' emotional wellbeing and consists of two subscales: positive affect (PA) and negative affect (NA). Each subscale contains 10 items, making a total of 20 items. Responses are rated on a 5-point Likert scale, ranging from 1 (“very slightly or not at all”) to 5 (“extremely”). A higher PA score relative to the NA score indicates greater positive emotional experiences. To compute the overall SWB score, the standardized life satisfaction score is added to the standardized positive affect score, and the standardized negative affect score is subtracted (Sheldon and Elliot, 1999).

### Statistical analysis

This study utilized SPSS statistical software for data analysis, following these specific steps. First, descriptive statistical analysis was conducted to calculate the means, standard deviations, and distribution characteristics of each variable, providing an overview of the dataset. Second, Pearson correlation analysis was performed to examine the fundamental relationships among PA, RT, MDS, and SWB, serving as a theoretical basis for subsequent mediation analysis. For the mediation effect analysis, this study employed the PROCESS macro (Hayes, 2013) and used Model 6 to test the chain mediation effect, specifically exploring how PA influences SWB through the sequential effects of RT and MDS. To ensure the robustness of the results, the Bootstrap method was applied with 5,000 resampling iterations, and 95% confidence intervals (CI) were calculated. If the confidence interval does not include zero, the mediation effect is considered statistically significant. Additionally, to assess the model fit, this study examined the explained variance (R^2^), standardized path coefficients (β), and significance levels (*p*-values) to confirm the statistical validity of the model. These analyses provide a scientific and reliable empirical basis for investigating the relationships among PA, RT, MDS, and SWB in college students.

### Reliability and validity

The reliability analysis of this study demonstrates high internal consistency for all measurement scales. Table 2 presents the structure, dimensions, and internal consistency (Cronbach's α) for each of the measurement scales used in the study.

**Table 2:** Measurement scales summary.

**Scale name**	**Dimensions**	**Items**	**Cronbach's α**	**Reference**
PARS-3	Intensity; duration; frequency.	3	-	Liang, 1994
RRS	Symptomatic rumination; compulsive thinking; reflective pondering.	22	0.942. symptomatic: 0.940; compulsive: 0.880; reflective: 0.875.	Nolen-Hoeksema, 1991; Han et al., 2007
FFMQ	Observing; describing; nonjudging; nonreactivity to inner experience; acting with awareness.	39	0.958. observing: 0.942; describing: 0.936; nonjudging: 0.939; nonreactivity: 0.908; awareness: 0.930.	Baer et al., 2006; Deng et al., 2011
SWB	Life satisfaction; positive affect; negative affect.	25	0.942. SWLS: 0.925; PANAS-positive: 0.951; PANAS-negative: 0.950.	Diener et al., 1985; Watson et al., 1988

The Cronbach's α coefficients for the key variables are as follows: RT = 0.942 (symptomatic rumination, 0.940; compulsive thinking, 0.880; reflective pondering, 0.875), MDS = 0.958(observing, 0.942; describing, 0.936; nonjudging, 0.939; nonreactivity to inner experience, 0.908; acting with awareness, 0.930), and SWB = 0.942(SWLS, 0.925; PANAS-positive, 0.951; PANAS-negative, 0.950). These values indicate excellent internal consistency, meeting the standard requirements for scale reliability. To assess construct validity, confirmatory factor analysis (CFA) was conducted using AMOS 26.0. A first-order three-factor model was tested, in which the three latent constructs-RT, MDS, and SWB-were each measured using their full set of item leve responses (RRS:22 items; FFMQ:39 items; SWLS+PANAS:25 items). The results showed that the model fit was excellent, with χ^2^/df = 1.202, which is well below the recommended threshold of 5.000, indicating a well-fitting model. Additionally, the fit indices were all above the acceptable threshold of 0.900: CFI = 0.989, NFI = 0.937, GFI = 0.915, TLI = 0.989, and RFI = 0.935. These results further confirm the robustness of the model's structure. Moreover, the root mean square error of approximation (RMSEA) = 0.014, which is significantly lower than the recommended threshold of 0.080, suggesting minimal model error and an optimal fit. Overall, these findings indicate that the measurement model in this study demonstrates strong construct validity and high reliability, ensuring the robustness and accuracy of the psychometric assessments.

## Results

### Common methodology bias control and testing

Given that the data in this study were collected through self-reported questionnaires, there is a possibility of common method bias (CMB) (Zhou and Long, 2004). To assess its impact, Harman's single-factor test was conducted. The results indicated that ten factors had eigenvalues >1, with the first factor accounting for 39.427% of the total variance, which is below the critical threshold of 40%. This suggests that common method bias is not a serious concern in this study.

### Descriptive statistics and correlation analysis

Table 3 presents the means, standard deviations, and correlation analysis results of the study variables (*N* = 1,075). The results indicate that the mean score of PA was M = 1.80 (SD = 0.86), RT had a mean score of M = 35.35 (SD = 9.28), MDS had a mean score of M = 103.87 (SD = 21.09), and SWB had a mean score of M = 74.57 (SD = 17.30).

**Table 3:** Variable means, standard deviations, and correlations (*N* = 1,075).

**Variables**	**M**	**SD**	**1**	**2**	**3**	**4**
Physical activity	1.80	0.86	1			
Rumination	35.35	9.28	−0.441^**^	2		
Mindfulness	103.87	21.09	0.452^**^	−0.482^**^	3	
Subjective wellbeing	74.57	17.30	0.368^**^	−0.388^**^	0.431^**^	4

^*^p < 0.05,^**^p < 0.01.

The PA variable used here is a 3-level categorical variable coded as 1 = low, 2 = moderate, 3 = high physical activity level. ^*^indicates statistical significance at the *p* < 0.05 level.

The correlation analysis showed that PA was significantly negatively correlated with RT (r = −0.441, *p* < 0.01), indicating that individuals with higher levels of PA were less likely to engage in RT. Additionally, PA was significantly positively correlated with MDS (r = 0.452, *p* < 0.01), suggesting that individuals who exercised more frequently were more likely to maintain focus and awareness in daily life. Furthermore, a significant positive correlation was found between PA and SWB (r = 0.368, *p* < 0.01), indicating that individuals who engaged in more PA reported higher levels of wellbeing. Further analysis revealed that RT was significantly negatively correlated with MDS (r = −0.482, *p* < 0.01), meaning that individuals who engaged in frequent negative thinking tended to have lower levels of present-moment awareness and acceptance. Likewise, RT was significantly negatively correlated with SWB (r = −0.388, *p* < 0.01), suggesting that a higher tendency for RT may reduce individuals' overall wellbeing. Additionally, MDS was significantly positively correlated with SWB (r = 0.431, *p* < 0.01), indicating that individuals with higher MDS levels generally reported a greater sense of wellbeing. These findings support the positive role of MDS in mental health and suggest that enhancing MDS levels may contribute to improving wellbeing.

### Regression analysis

Table 4 presents the direct, indirect, and total effects of PA on SWB, while also examining the mediating roles of RT and MDS. The results indicate that PA not only directly enhances SWB but also exerts an indirect effect through reducing RT and increasing MDS.

**Table 4:** Analysis of regression relationships between variables.

**Effect type**	**Path**	**B**	**SE**	**t**	***p***	**95% Confidence interval**	
						**LLCI**	**ULCI**
Direct effect	PA → SWB	0.167	0.054	3.09	0.002	0.061	0.273
Indirect effect	PA → RT	−4.751	0.295	−16.113	0.0	−5.329	−4.172
	PA → MDS	7.264	0.695	10.454	0.0	5.901	8.682
	RT → MDS	−0.796	0.065	−12.332	0.0	−0.923	−0.669
	RT → SWB	−0.027	0.005	−5.204	0.0	−0.037	−0.017
	MDS → SWB	0.01	0.002	4.425	0.0	0.006	0.014
Total effect	PA → SWB	0.405	0.048	8.472	0.0	0.311	0.498

Unstandardized regression coefficients (B) are reported. PA, Physical Activity; RT, Rumination; MDS, Mindfulness; SWB, Subjective WellBeing. The regression model predicting SWB explained 12.2% of the variance (R^2^ = 0.122).

The regression analysis demonstrated that PA had a significant direct effect on SWB (B = 0.167, *p* = 0.002), suggesting that individuals with higher levels of PA tend to report greater wellbeing. However, further analysis revealed that PA also influences SWB indirectly through a chain mediation effect involving RT and MDS. Specifically, PA was significantly negatively associated with RT (B = −4.751, *p* < 0.001), indicating that individuals who engage in more PA are less likely to fall into negative thinking patterns. Additionally, PA was significantly positively associated with MDS (B = 7.264, *p* < 0.001), suggesting that regular exercise contributes to enhanced awareness and a greater focus on present experiences. Moreover, the results showed that RT had a significant negative effect on MDS (B = −0.796, *p* < 0.001), implying that individuals who engage in persistent negative thinking are less likely to maintain a mindful state. Similarly, RT had a significant negative effect on SWB (B = −0.027, *p* < 0.001), indicating that excessive focus on negative events may diminish overall wellbeing. In contrast, MDS had a significant positive effect on SWB (B = 0.010, *p* < 0.001), suggesting that individuals with higher MDS levels tend to experience greater wellbeing. Further examination of the total effect revealed that PA had a significant overall effect on SWB (B = 0.405, *p* < 0.001), which was notably greater than the direct effect (B = 0.167). This finding highlights the critical role of the indirect pathways in the relationship between PA and SWB. Specifically, PA enhances SWB not only through its direct effects but also by reducing RT and increasing MDS, which further contribute to greater wellbeing. The regression model predicting SWB demonstrated a modest explanatory power, accounting for 12.2% of the variance (R^2^ = 0.122).

### Mediation effect analysis

The Table 5 presents the mediation effect analysis of PA on SWB through RT and MDS.

**Table 5:** Analysis of the mediating effect of physical activity and subjective wellbeing.

**Path**	**B**	**Boot SE**	**95% Confidence interval**		**Proportion of mediation in the total effect**
			**Lower limit**	**Upper limit**	
PA → RT → SWB	0.127	0.027	0.075	0.18	31%
PA → MDS → SWB	0.073	0.02	0.036	0.113	18%
PA → RT → MDS → SWB	0.038	0.01	0.073	0.06	9%

PA, Physical activity; RT, Rumination; MDS, Mindfulness; SWB, Subjective wellbeing.

First, the mediation effect of PA → RT → SWB was 0.127, accounting for 31% of the total effect, with a Bootstrap confidence interval of [0.075, 0.18], indicating that this mediation pathway was significant. This suggests that PA can enhance SWB by reducing RT, and this pathway contributes a substantial proportion to the total effect. Second, the mediation effect of PA → MDS → SWB was 0.073, accounting for 18% of the total effect, with a Bootstrap confidence interval of [0.036, 0.113], which also reached significance. This indicates that PA indirectly enhances SWB by increasing MDS, suggesting that exercise helps individuals develop greater present-moment awareness, which in turn improves wellbeing. Finally, the chain mediation effect of PA → RT → MDS → SWB was 0.038, accounting for 9% of the total effect, with a Bootstrap confidence interval of [0.073, 0.06]. Although this effect contributed a smaller proportion compared to the other mediation pathways, it remained significant. This finding suggests that PA reduces RT, which subsequently enhances MDS, ultimately leading to higher SWB. It highlights that reducing RT not only directly improves wellbeing but also facilitates MDS, forming a more complex indirect influence pathway.

**Figure 2:**
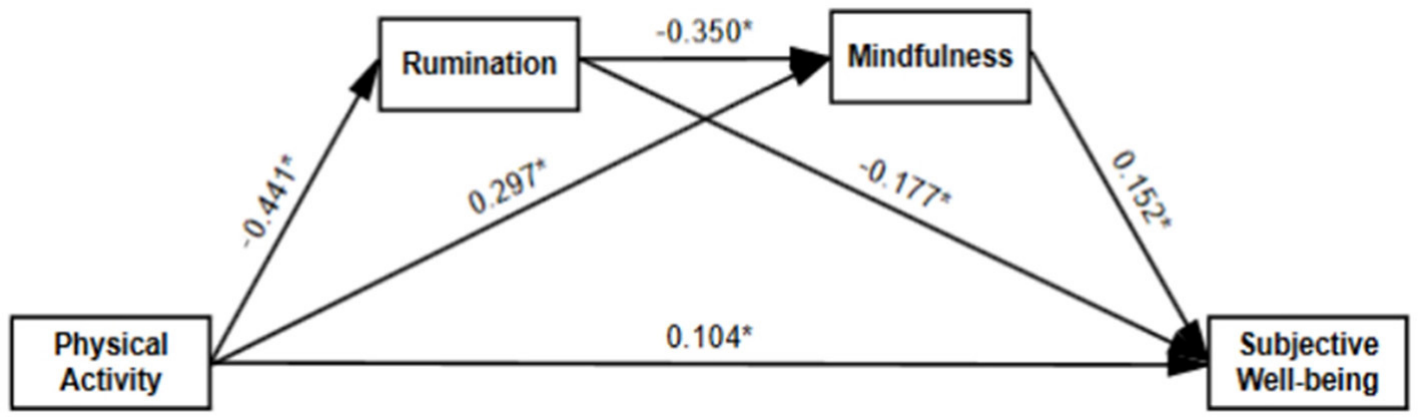
A chain-mediated model of PA and SWB. *P < 0.05.

Figure 2 illustrates the chain mediation model, in which PA influences SWB through RT and MDS. The results supported all the proposed hypotheses, as all path coefficients reached statistical significance (*p* < 0.05). First, PA had a significant positive direct effect on SWB (β = 0.104, *p* < 0.05), indicating that higher levels of PA directly enhance individuals' wellbeing. However, further analysis revealed that PA also influenced SWB through indirect pathways, which accounted for a substantial proportion of the total effect. In the first indirect pathway, PA significantly negatively predicted RT (β= −0.441, *p* < 0.05), suggesting that individuals with higher PA levels were less likely to engage in negative thinking patterns. Additionally, RT significantly negatively predicted SWB (β= −0.177, *p* < 0.05), indicating that higher levels of RT were associated with lower SWB. This pathway confirmed that PA enhances wellbeing by reducing RT (PA → RT → SWB). In the second indirect pathway, PA significantly positively predicted MDS (β= 0.297, p <0.05), meaning that individuals who engage in more PA tend to maintain greater awareness of the present moment. Furthermore, MDS had a significant positive effect on SWB (β= 0.152, *p* < 0.05), suggesting that individuals with higher MDS levels generally report greater wellbeing. This pathway demonstrated that PA enhances SWB by increasing MDS (PA → MDS → SWB). Additionally, the study further explored the chain mediation effect, in which PA reduces RT, which in turn enhances MDS, ultimately improving SWB (PA → RT → MDS → SWB). All path coefficients in this chain mediation pathway were statistically significant, with RT significantly negatively predicting MDS (β= −0.350, *p* < 0.05), indicating that individuals with lower RT levels are more likely to maintain a mindful state.

## Discussion

### The relationship between physical activity and subjective wellbeing

This study found a significant positive correlation between PA and SWB, indicating that individuals with higher levels of PA report greater happiness. This result supports the theoretical framework that PA promotes SWB through both physiological and psychological pathways (Guo et al., 2024; Buecker et al., 2021)^.^ From a physiological perspective, PA regulates emotions by activating the dopaminergic reward pathway and promoting the secretion of endorphins and serotonin (Chen and Nakagawa, 2023). Dopamine enhances behavioral motivation and pleasure, endorphins alleviate pain and induce euphoria, while serotonin stabilizes mood and reduces anxiety levels (Mikkelsen et al., 2017)^.^ Moreover, long-term exercise induces neurogenesis in the hippocampus and synaptic plasticity in the prefrontal cortex, thereby improving stress adaptation (Soya et al., 2011). For instance, Erickson et al. found that 6 months of aerobic exercise increased hippocampal volume by 2% and was significantly associated with a reduction in depressive symptoms (Erickson et al., 2011). In addition to physiological mechanisms, psychological and social cognitive factors also play crucial roles in this process. From a psychological perspective, PA indirectly enhances SWB by strengthening self-efficacy and social connectedness (Paxton et al., 2010). According to Bandura's self-efficacy theory, regular exercise enables individuals to gradually develop a positive belief in their own abilities through the achievement of fitness goals. This enhancement of self-efficacy can generalize to other domains of life, ultimately improving overall self-esteem (Bandura and Wessels, 1997; Voskuil and Robbins, 2015)^.^ Meanwhile, physical activities are often accompanied by social interactions, which not only alleviate loneliness but also reinforce a sense of belonging and social support, further consolidating wellbeing (Zhang et al., 2024). Therefore, PA is not only a means of maintaining physiological health but also a multidimensional intervention strategy that integrates physiological and psychological mechanisms, ultimately enhancing an individual's overall SWB in a systematic manner.

### The mediating role of rumination

This study found that RT plays a partial mediating role in the relationship between PA and SWB, accounting for 31% of the total effect. This indicates that PA not only directly influences SWB but also indirectly enhances wellbeing by reducing RT. RT, characterized by persistent negative focus on adverse events (Nolen-Hoeksema et al., 2008), is neurobiologically associated with excessive activation of the default mode network (DMN) and insufficient prefrontal regulation (Hamilton et al., 2015). This study identified three primary pathways through which PA inhibits RT: First, during PA, individuals' attentional resources are directed toward bodily sensations (e.g., breathing rhythm, muscle movement) and goal-oriented tasks (e.g., movement execution), effectively interrupting sustained focus on negative experiences (de Bruin et al., 2016). Neuroimaging studies have shown that exercise reduces excessive DMN activation (Zhang et al., 2024), which is closely linked to RT (Hamilton et al., 2015), while enhancing task-related neural networks such as the prefrontal-parietal control network, responsible for goal-directed attention allocation. Increased activation of this network suppresses self-referential processing within the DMN, thereby reducing RT tendencies (de Bruin et al., 2016). Second, PA modulates the hypothalamic—pituitary—adrenal (HPA) axis, lowering cortisol levels under chronic stress (Puterman et al., 2011), thereby alleviating physiological stress responses and weakening emotional sensitivity to negative events, preventing individuals from falling into the “rumination-emotional deterioration” cycle. Third, according to Behavioral Activation Theory (BAT), PA reinforces goal-directed behaviors, thus breaking the passivity associated with RT (Lyubomirsky et al., 2015; Kanter et al., 2010). RT is often accompanied by low motivation and low energy levels, whereas exercise increases behavioral activation, disrupting this pattern. For instance, achieving exercise-related goals enhances self-efficacy, shifting individuals from passive RT to proactive engagement in activities (Jeong et al., 2023). These mechanisms collectively suggest that PA not only regulates emotions through physiological pathways but also systematically enhances SWB by intervening in cognitive processes such as reducing RT, providing new insights into the relationship between PA and mental health.

### The mediating role of mindfulness

This study found that MDS plays a significant mediating role in the relationship between PA and SWB, indicating that individuals with higher levels of PA tend to exhibit greater MDS, which in turn enhances wellbeing. MDS, defined as the ability to focus on present experiences with a non-judgmental attitude, has been widely recognized as a key protective factor for mental health (Chems-Maarif et al., 2025). The association between PA and MDS can be explained from multiple perspectives. First, certain forms of exercise, such as yoga and Tai Chi, inherently require individuals to maintain continuous attention on breathing and bodily movements. This synchronization of “body awareness” directly reinforces the core characteristics of MDS (Hawkes, 2012). Second, neuroscience research suggests that the prefrontal cortex exerts top-down regulation to inhibit excessive amygdala activation, a mechanism particularly prominent in MDS training. PA has been shown to enhance prefrontal cortex function, a brain region closely related to emotional regulation and cognitive control (Kramer and Erickson, 2007). Strengthening this function helps suppress excessive emotional responses from the amygdala, thereby promoting greater awareness and acceptance of emotions. Furthermore, the impact of MDS on wellbeing can be understood in two key ways. First, individuals with high MDS are better able to recognize and immerse themselves in positive emotional experiences while reducing fixation on negative emotions (Garland et al., 2015). Second, MDS enhances psychological resilience, enabling individuals to recover more quickly from stress and restore emotional stability (Oh et al., 2022). These findings suggest that MDS is not only a psychological mechanism underlying the effects of PA but also a crucial cognitive bridge connecting PA to enhanced wellbeing.

### The chain-mediating role of rumination and mindfulness

This study found that RT and MDS form a chain-mediating pathway in the relationship between PA and SWB, indicating that PA reduces RT, which in turn enhances MDS, ultimately leading to improved wellbeing. RT, characterized by a cognitive pattern of negative self-focus, exacerbates individuals' repetitive thinking about negative events (Nolen-Hoeksema et al., 2008), and its neural basis is closely related to excessive activation of the DMN (Hamilton et al., 2015). PA disrupts this pattern through dual mechanisms: first, exercise requires attention to be focused on bodily movements, and this outward allocation of attentional resources directly suppresses DMN activity, thereby reducing RT (Brewer et al., 2011); second, exercise enhances the functional connectivity of the prefrontal-limbic circuit, improving cognitive control and making it easier for individuals to shift from a ruminative state to a mindful “present-moment awareness” mode (Tang et al., 2015). Furthermore, the reduction of RT provides cognitive space for the cultivation of MDS. When individuals are no longer occupied by negative thoughts, they are more likely to observe and accept present experiences with an open attitude. For example, intervention studies combining PA with MDS training indicate that individuals who engage in both running and attentional awareness exercises exhibit significantly greater improvements in MDS than those in a single-modality training group (Gotink et al., 2016). This chain-mediating mechanism reveals a dual-pathway effect of PA on mental health, where it reduces negative cognition (RT) while enhancing positive regulation (mindfulness), providing theoretical support for the design of exercise-based psychological interventions.

### Practical implications

The findings of this study have multiple practical implications. First, they provide empirical evidence for public health policymakers, emphasizing the integration of physical exercise into mental health promotion systems. Second, they suggest that mental health interventions could incorporate both PA and MDS training, such as embedding exercise goal-setting in Cognitive Behavioral Therapy or integrating physical activities (e.g., dynamic meditation) into Mindfulness-Based Stress Reduction (MBSR) programs. Additionally, for specific populations (e.g., individuals with high rumination tendencies or depression), a “PA-MDS” stepped intervention model could be designed: in the initial phase, aerobic exercise can be used to reduce RT frequency, followed by MDS training to reinforce emotional regulation skills in later stages. Furthermore, research indicates that such integrated interventions are more effective than standalone approaches. For example, Torre et al. found that compared to engaging in either exercise or MDS training alone, combining MDS with aerobic exercise led to simultaneous improvements in emotional stability and life satisfaction, resulting in greater overall psychological wellbeing (Torre et al., 2023).

### Strengths and limitations

This study reveals multiple pathways through which PA enhances SWB: it directly improves emotional states and also indirectly influences wellbeing through a chain-mediating mechanism of reducing RT → enhancing MDS. These findings expand the theoretical framework of exercise psychology, demonstrating that PA not only alleviates negative cognition (e.g., rumination) but also strengthens positive psychological resources through MDS cultivation. Future research could build on these insights to design integrated exercise-cognition interventions, such as developing aerobic exercise combined with MDS training for individuals with high RT tendencies or creating digital MDS applications incorporating PA elements to enhance the precision and personalization of mental health interventions. However, one limitation of this study is its reliance on self-report questionnaires, which may be susceptible to biases such as social desirability and recall errors. These biases may potentially compromise the accuracy of the participants' responses. Future research is encouraged to incorporate behavioral indicators, physiological measures, or longitudinal designs to improve objectivity and strengthen causal inference.

## Conclusion

This study demonstrated that PA significantly enhances SWB through three distinct pathways: direct emotional regulation, reduction of RT, and enhancement of MDS. Statistical analyses confirmed the significance of both direct and indirect effects, with a chain-mediated pathway from PA to RT to MDS to SWB. Notably, reduced RT was found to free up cognitive resources, facilitating the development of MDS and producing a synergistic “cognitive decoupling–MDS gain” effect. From a theoretical perspective, these findings enrich the understanding of the cognitive-emotional mechanisms underlying the benefits of PA on mental health. Practically, the results support the design of integrated PA and mindfulness-based interventions—such as group exercise programs combined with MDS-oriented social skills training—for improving student mental health, especially in populations vulnerable to anxiety and negative thinking patterns. Future studies may also explore neurobiological mechanisms, including the potential moderating role of neuroplasticity biomarkers like brain-derived neurotrophic factor (BDNF).

## Data Availability

The original contributions presented in the study are included in the article/supplementary material, further inquiries can be directed to the corresponding author.
